# The PREgnancy and FERtility (PREFER) Study Investigating the Need for Ovarian Function and/or Fertility Preservation Strategies in Premenopausal Women With Early Breast Cancer

**DOI:** 10.3389/fonc.2021.690320

**Published:** 2021-06-03

**Authors:** Eva Blondeaux, Claudia Massarotti, Valeria Fontana, Francesca Poggio, Luca Arecco, Piero Fregatti, Claudia Bighin, Irene Giannubilo, Tommaso Ruelle, Maria Grazia Razeti, Luca Boni, Paola Anserini, Lucia Del Mastro, Matteo Lambertini

**Affiliations:** ^1^ Department of Internal Medicine and Medical Sciences (DiMI), School of Medicine, University of Genova, Genova, Italy; ^2^ Breast Unit, IRCCS Ospedale Policlinico San Martino, Genova, Italy; ^3^ Physiopathology of Human Reproduction Unit, IRCCS Ospedale Policlinico San Martino, Genova, Italy; ^4^ Clinical Epidemiology Unit, IRCCS Ospedale Policlinico San Martino, Genova, Italy; ^5^ Department of Medical Oncology, U.O. Clinica di Oncologia Medica, IRCCS Ospedale Policlinico San Martino, Genova, Italy; ^6^ U.O.C. Clinica di Chirurgia Senologica, Department of Surgery, IRCCS Ospedale Policlinico San Martino, Genova, Italy; ^7^ U.O.C. Oncologia Medica 2, Department of Medical Oncology, Ospedale Policlinico San Martino, Genova, Italy

**Keywords:** breast cancer, premenopausal patients, premature ovarian insufficiency, fertility preservation, gonadotoxicity

## Abstract

**Background:**

Offering ovarian function and/or fertility preservation strategies in premenopausal women with newly diagnosed breast cancer candidates to undergo chemotherapy is standard of care. However, few data are available on uptake and main reasons for refusing these options.

**Methods:**

The PREFER study (NCT02895165) is an observational, prospective study enrolling premenopausal women with early breast cancer, aged between 18 and 45 years, candidates to receive (neo)adjuvant chemotherapy. Primary objective is to collect information on acceptance rates and reasons for refusal of the proposed strategies for ovarian function and/or fertility preservation available in Italy.

**Results:**

At the study coordinating center, 223 patients were recruited between November 2012 and December 2020. Median age was 38 years (range 24 – 45 years) with 159 patients (71.3%) diagnosed at ≤40 years. Temporary ovarian suppression with gonadotropin-releasing hormone agonists (GnRHa) was accepted by 58 out of 64 (90.6%) patients aged 41-45 years and by 151 out of 159 (95.0%) of those aged ≤40 years. Among patients aged ≤40 years, 57 (35.8%) accepted to access the fertility unit to receive a complete oncofertility counseling and 29 (18.2%) accepted to undergo a cryopreservation technique. Main reasons for refusal were fear of delaying the initiation of antineoplastic treatments and contraindications to the procedure or lack of interest in future childbearing. Patients with hormone-receptor positive breast cancer had a tendency for a higher acceptance rates of ovarian function and/or fertility preservation strategies than those with hormone-receptor negative disease.

**Conclusions:**

More than 90% of premenopausal women with early breast cancer, and particularly those with hormone receptor-positive disease, were concerned about the potential risk of chemotherapy-induced premature ovarian insufficiency and/or infertility and accepted GnRHa administration. Less than 1 out of 5 women aged ≤40 years accepted to undergo cryopreservation strategies.

## Introduction

Among women of reproductive age, breast cancer is the most frequent diagnosed malignancy (1). Chemotherapy still remains an important component of the care of many premenopausal breast cancer patients also taking into account their higher risk of developing more aggressive breast cancer subtypes (2, 3). The long-term side effects of chemotherapy including the potential damage to women’s ovarian function and fertility potential are of high concern for a significant proportion of women diagnosed during their reproductive age (4, 5). Two main approaches are available for trying to counteract the long-term side effects of chemotherapy on breast cancer patients’ reproductive health (6). Firstly, ovarian function preservation aims to reduce the potential long-term side effects of chemotherapy-induced premature ovarian insufficiency (POI) that include menopause-related symptoms, psychosocial issues and other health problems (4). This approach can be of importance also to patients not interested in future conception. Secondly, fertility preservation aims to increase the chances of achieving a post-treatment pregnancy in patients willing to complete their family plan after breast cancer treatment (7–10).

Current guidelines recommend to perform a complete oncofertility counseling to all premenopausal women at the time of cancer diagnosis (7–10). During this counseling, the potential risk of chemotherapy-induced POI and subsequent possible impaired ovarian function and fertility should be discussed, and patients interested in avoiding these side effects are offered the available strategies for preserving ovarian function and/or fertility (7–10). In premenopausal breast cancer patients, temporary ovarian suppression with gonadotropin-releasing hormone agonists (GnRHa) during chemotherapy is considered as standard strategy for ovarian function preservation (7–10). Available strategies for fertility preservation include cryopreservation of embryos, oocytes and/or ovarian tissue to be preferably proposed to women diagnosed at ≤40 years (≤36 years for ovarian tissue cryopreservation) considering the low success rate in older patients (7–10). Despite the widespread use of these strategies among breast cancer patients, few data are available on the uptake and on the main reasons for refusal of these options.

The prospective PREgnancy and FERtility (PREFER) study aims to investigate the actual needs and preferences of patients regarding the proposed options for ovarian function and/or fertility preservation available in Italy (11). Previous results of the PREFER study indicated that a significant proportion of young women with newly diagnosed breast cancer are concerned about the possible risk of chemotherapy-induced POI and/or infertility but only 12% decide to undergo the proposed cryopreservation procedures (12). Here, we present updated results from the PREFER study.

## Methods

### Study Design and Participants

Details of the PREFER study design and methods were previously reported (11, 12). Briefly, this is an ongoing multicenter prospective observational study aiming to optimize care and improve knowledge on ovarian function and/or fertility preservation in premenopausal women with early breast cancer.

The study includes premenopausal women with early breast cancer aged between 18 and 45 years who are candidates to undergo (neo)adjuvant chemotherapy. Exclusion criteria are *de novo* metastatic disease, prior exposure to chemotherapy and/or radiation therapy and severe psychiatric disorders.

In the present analysis, we updated previously reported data of patients included at the coordinating center (12). Moreover, further analyses were conducted to explore the impact of breast cancer hormone-receptor status on patients’ choices. In addition, preliminary efficacy results of cryopreservation strategies in terms of number of retrieved and cryopreserved oocytes and response rate to controlled ovarian stimulation are reported.

Due to the slow opening of the other Italian participating centers, we believe that the current updated analysis can provide additional important information before the possibility to analyze the data from all centers, expected to occur in 2 years from now.

All patients provided a written informed consent before study entry. The study was approved by the Ethics Committee of the coordinating center in November 2012.

The study is registered on ClinicalTrials.gov (NCT02895165).

### The PREFER Algorithm for the Oncofertility Counseling

**Figure 1:**
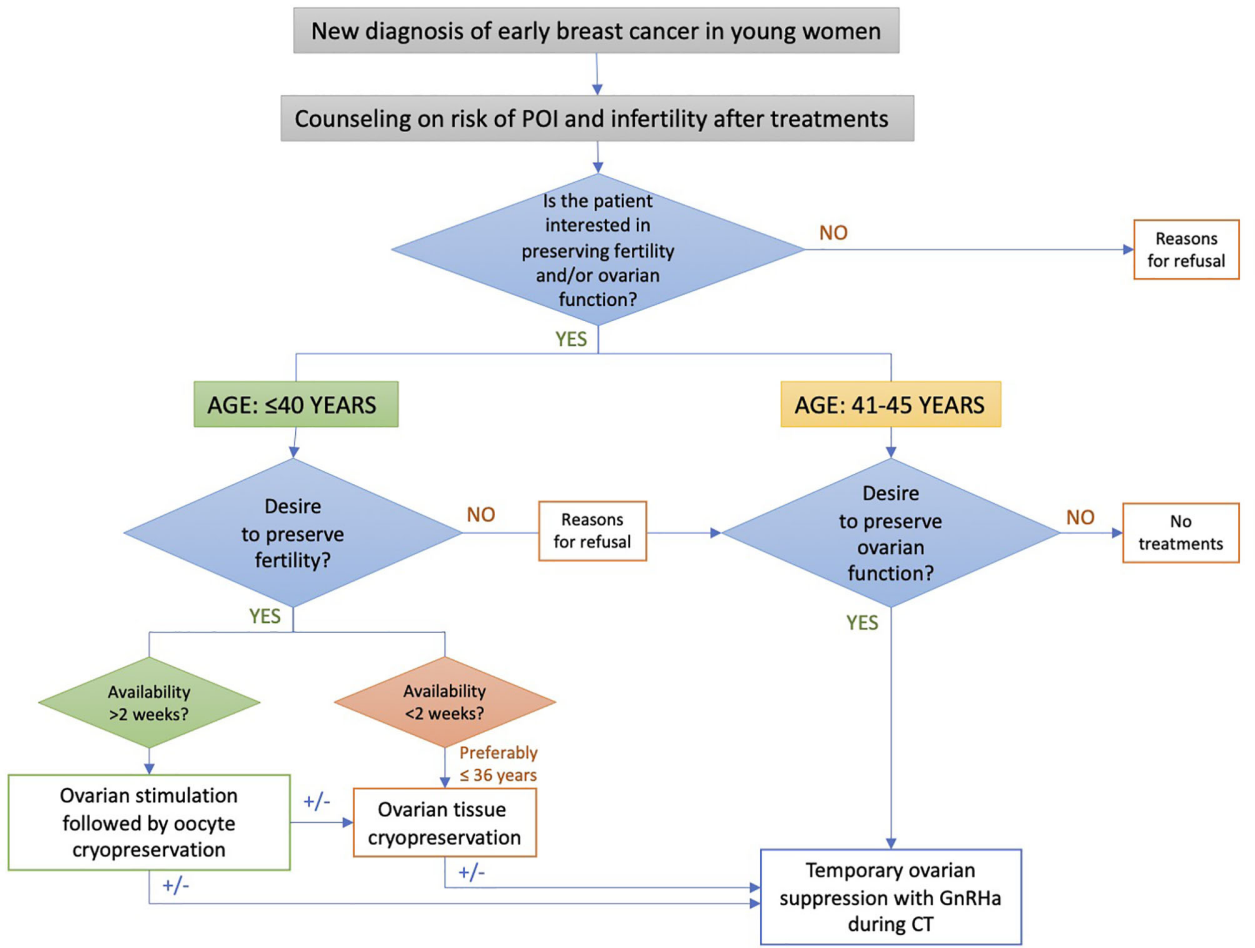
Oncofertility counseling algorithm for patients enrolled in the PREFER study. POI, premature ovarian insufficiency; GnRHa, gonadotropin-releasing hormone agonist.

A specific algorithm was developed to implement a proper oncofertility counseling (11, 12) (**Figure 1**). Briefly, the risk of chemotherapy-induced POI and/or infertility and the available strategies for ovarian function and/or fertility preservation are discussed by the oncologist with all premenopausal patients as soon as possible after diagnosis and before starting any systemic anti-cancer treatment. For patients diagnosed at ≤40 years and interested in fertility preservation, a complete oncofertility counseling with a fertility specialist is offered and both oocyte and/or ovarian tissue cryopreservation are proposed according to the time available before anticancer treatment initiation. Notably, in Italy, embryo cryopreservation is prohibited by law in these patients. After cryopreservation techniques are performed, temporary ovarian suppression with GnRHa during chemotherapy is offered. Whereas, for patient diagnosed between 41 and 45 years and interested in ovarian function preservation, only temporary ovarian suppression with GnRHa during chemotherapy is offered. Cryopreservation strategies are not proposed in this age group due to their low success rate in breast cancer patients older than 40 years (13).

### Study Objectives and Statistical Analysis

Primary objective of the PREFER study is to assess patients’ preferences and choices of the different available strategies for ovarian function and/or fertility preservation, in terms of acceptance rate, and to collect the reasons for refusal of the proposed strategies. To investigate the efficacy of cryopreservation strategies in patients aged ≤40 years at diagnosis is a secondary objective. Potential differences in acceptance rates of ovarian function and/or fertility preservation strategies according to hormone-receptor status were explored.

Patient, tumor and treatment characteristics, types of strategies for ovarian function and/or fertility preservation offered and accepted by patients, and reasons for refusal are prospectively collected in electronic case report forms. Statistical analyses are mainly descriptive. Means and standard deviations were used to summarize continuous variables, whereas counts and percentages were used for categorical variables. Statistical analyses were performed using SAS 9.2 (SAS Institute).

## Results

From November 2012 to December 2020, 223 consecutive newly diagnosed premenopausal breast cancer patients were included at the Breast Unit of the coordinating center.

Baseline characteristics are reported in **Table 1**. Median age at study entry was 38 years (range 24-45). A total of 64 (28.7%) women were diagnosed between the age of 41 and 45 years and 159 (71.3%) at ≤40 years. At the time of breast cancer diagnosis, 150 (67.3%) patients had at least one child.

**Table 1:** Baseline characteristics of the patients included in the PREFER study.

Characteristics	Total cohort N = 223 No. (%)
**Age, median (range), years**	38 (24.0-45.0)
**Age distribution, characteristics**	
≤ 40	159 (71.3)
41 - 45	64 (28.7)
**Previous pregnancy**	150 (67.3)
**Number of previous pregnancies, median (range)**	2 (1-6)
**Partner at breast cancer diagnosis**	
Present with stable relationship	158 (70.9)
No partner present	58 (26.0)
Other	7 (3.1)
**Tumor size**	
≤ 2 cm	101 (45.3)
> 2 cm	121 (54.3)
Unknown	1 (0.5)
**Nodal status**	
Node negative	96 (43.1)
Node positive	122 (54.7)
Unknown	5 (2.2)
**Hormone receptor status**	
ER-positive and/or PgR-positive	173 (77.6)
ER-negative and PgR-negative	50 (22.4)
**HER2 status**	
Positive	76 (34.1)
Negative	147 (65.9)
**Timing of chemotherapy**	
Adjuvant	129 (57.9)
Neoadjuvant	92 (41.3)
Missing	2 (0.9)
**Type of chemotherapy**	
Anthracycline- and taxane-based	184 (82.5)
Others	37 (16.1)
Type of endocrine therapy*	
Tamoxifen ± GnRHa	59 (34.1)
Aromatase inhibitor + GnRHa	77 (44.5)
Tamoxifen ± GnRHa → Aromatase inhibitor + GnRHa	26 (15.0)
No endocrine therapy	1 (0.6)
Chemotherapy ongoing	7 (4.0)
Missing	1 (0.6)

*Percentages calculated on the total number of patients with hormone receptor positive disease (n = 173).

ER, estrogen receptor; PgR, progesterone receptor; GnRHa, gonadotropin-releasing hormone agonist.

**Figure 2:**
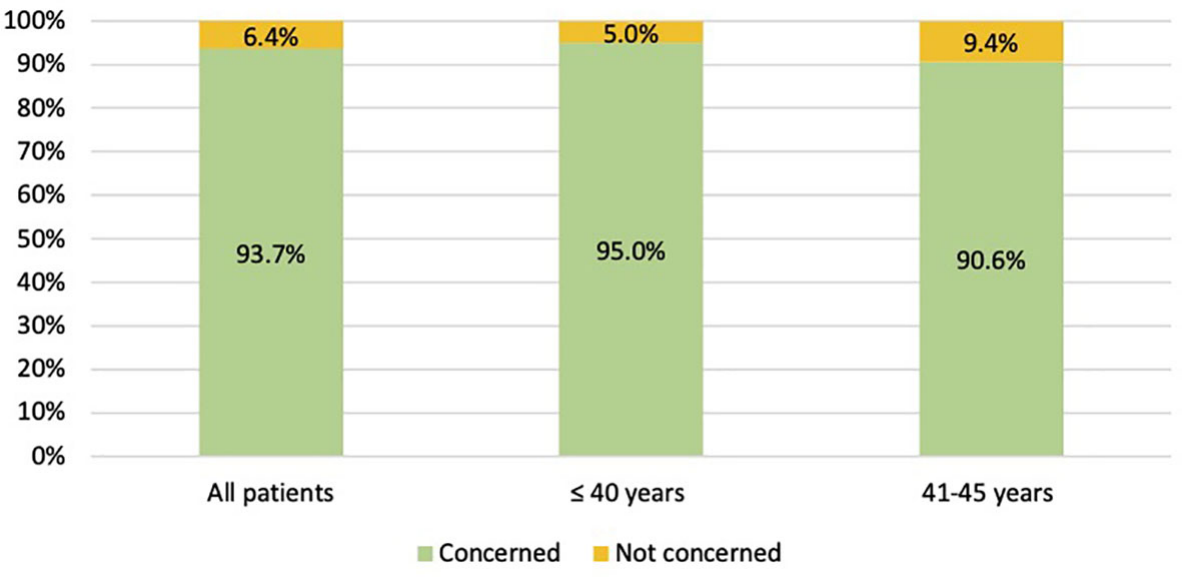
Number of patients concerned about the potential risk of chemotherapy-induced premature ovarian insufficiency AND/OR subsequent impaired fertility.

Overall, the majority of patients (209, 93.7%) was concerned about the potential risk of developing chemotherapy-induced POI and/or infertility (**Figure 2**). Specifically, 58 (90.6%) patients aged between 41 and 45 years and 151 (95.0%) aged ≤40 years were sensitive to these issues (**Figure 2**). For the 14 (6.3%) patients not concerned about potential risk of developing chemotherapy-induced POI and/or infertility, main reasons were lack of interest in ovarian function preservation in 10 patients (71.4%), prior completion of family planning in 3 (21.4%), and lack of interest in future childbearing in 1 (7.1%).

**Figure 3:**
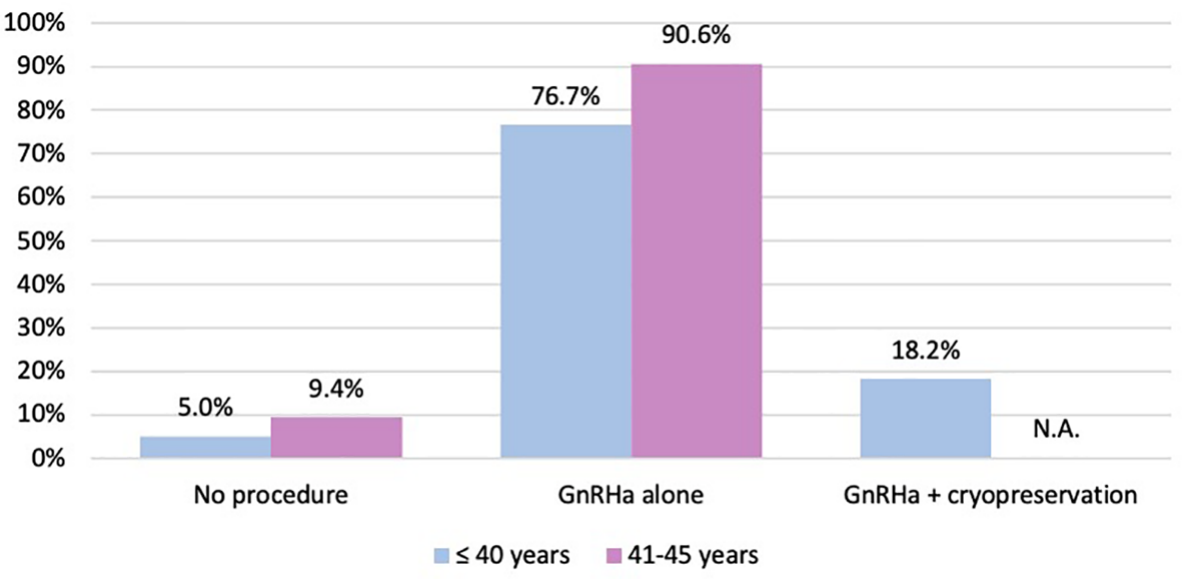
Number of patients who took active steps towards the offered strategies for ovarian function and/or fertility preservation. GnRHa, gonadotropin-releasing hormone agonist; NA, not applicable.

**Figure 4:**
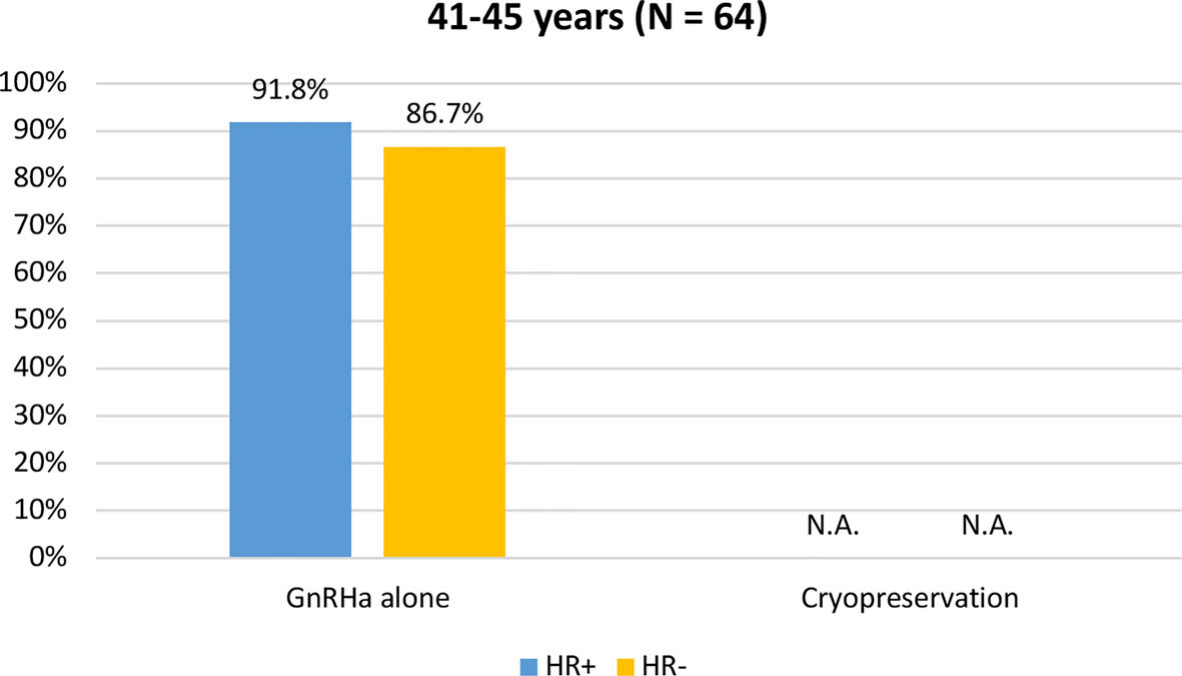
Acceptance rate of the offered strategies for ovarian function preservation according to hormonal receptor status in patients diagnosed between 41 and 45 years of age. GnRHa, gonadotropin-releasing hormone agonist; NA, not applicable.

Among the 64 patients diagnosed between 41 and 45 years of age, 58 (90.6%) were concerned about the possible chemotherapy-induced POI and accepted the use of temporary ovarian suppression with GnRHa during chemotherapy as a strategy for ovarian function preservation (**Figure 3**). Two patients diagnosed between 41 and 45 years underwent a complete reproductive counseling with the fertility specialists because of a strong pregnancy desire; however, none of them underwent a cryopreservation strategy. When assessing acceptance rates by hormone receptor status, active steps towards the offered strategy for ovarian function preservation with GnRHa administration were perused by 45 out of 49 (91.8%) women with hormone receptor-positive breast cancer and by 13 out of 15 (86.7%) with hormone receptor-negative disease (**Figure 4**).

Among the 159 patients aged ≤40 years at diagnosis, a complete reproductive counseling conducted at the fertility unit was accepted by 55 (34.6%) women. Among the 94 patients that refused to undergo a complete reproductive counseling, main reasons were previous completion of family planning in 63 (67.0%), concerns about a possible delay in cancer treatment in 11 (11.7%), lack of interest in future childbearing in 10 (10.6%) and oncological ineligibility for cryopreservation procedures in 5 (5.3%), unknown reason in 4 (4.3%) and availability of cryopreserved oocytes before breast cancer diagnosis in 1 (1.1%). Among the 55 patients that underwent a complete reproductive counseling by the fertility specialists, 5 (9.1%) were deemed medically ineligible by the fertility specialist to a cryopreservation technique mainly due to low ovarian reserve or high risk of complications, 21 (38.2%) refused the proposed cryopreservation strategies and 29 (52.7%) accepted to receive at least one of cryopreservation option. Specifically, among the 29 patients that accepted fertility preservation procedures, 24 underwent oocyte cryopreservation, 4 ovarian tissue cryopreservation, and one both oocyte and ovarian tissue cryopreservation. Among the 25 patients that underwent oocyte cryopreservation, median number of retrieved oocytes was 12 (range 0-42) and median number of cryopreserved oocytes was 9 (range 0-24). Poor response rate (i.e. retrieval of ≤4 oocytes) was observed in 3 out of 25 (12%) patients.

Among the 21 patients that refused the proposed cryopreservation strategies, main reasons were fear of delaying the initiation of antineoplastic treatments for 6 (28.8%), refusal of further medicalization after complete counseling (23.8%), lack of interest in the procedure after complete counseling for 6 (28.8%), and lack of support from a partner for 2 (9.5%), prior completion of family planning for 2 (9.5%).

Overall, among women aged ≤40 years at diagnosis, 151 (95.0%) took active steps towards the offered strategy for ovarian function and/or fertility preservation. The use of temporary ovarian suppression with GnRHa during chemotherapy was accepted by 122 (76.7%) women, while the use of cryopreservation strategies (followed by temporary ovarian suppression with GnRHa during chemotherapy) was accepted by 29 (18.2%) patients (**Figure 3**).

**Figure 5:**
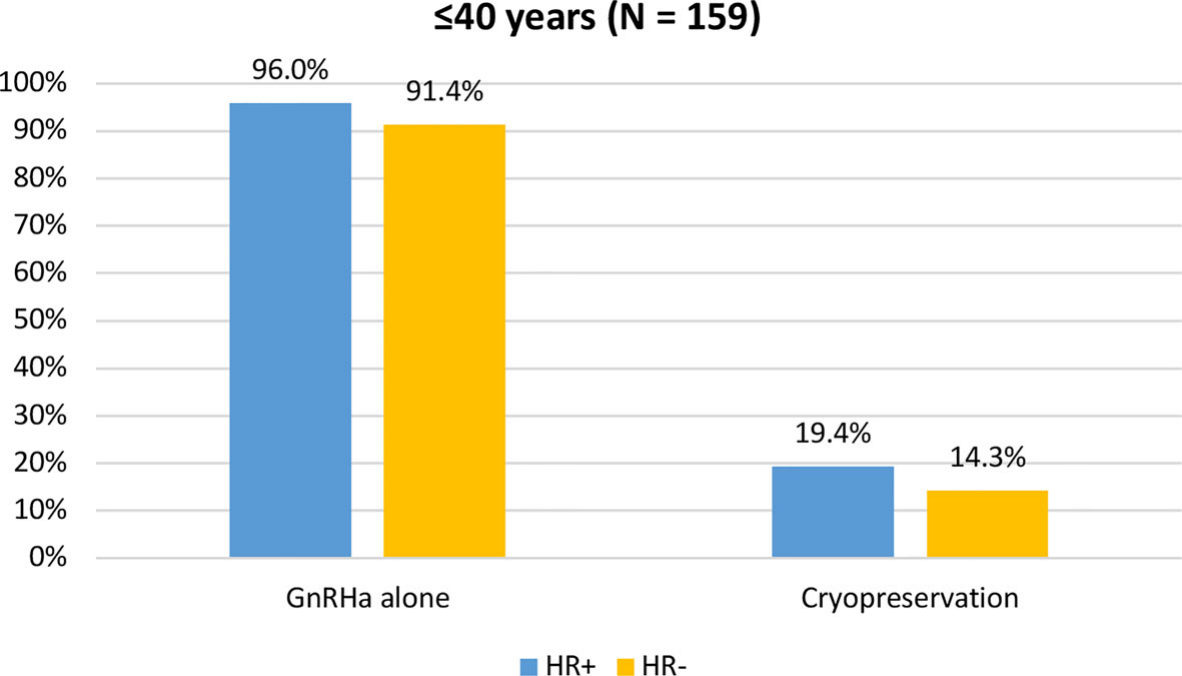
Acceptance rate of the offered strategies for ovarian function and/or fertility preservation according to hormonal receptor status in patients diagnosed at ≤40 years of age. GnRHa, gonadotropin-releasing hormone agonist.

When assessing acceptance rates by hormone receptor status, active steps towards the offered strategy for ovarian function and/or fertility preservation were pursued by 119 out of 124 (96%) women with hormone receptor-positive breast cancer and by 32 out of 35 (91.4%) with hormone receptor-negative breast cancer. In particular, the use of temporary ovarian suppression with GnRHa during chemotherapy was accepted by 119 (96%) women with hormone receptor-positive breast cancer and by 32 (91.4%) with hormone receptor-negative disease. Cryopreservation strategies were accepted by 24 (19.4%) women with hormone receptor-positive breast cancer and by 5 (14.3%) with hormone receptor-negative disease (**Figure 5**).

## Discussion

Although performing a complete oncofertility counseling to discuss the potential risk of chemotherapy-induced POI and infertility and to offer the available strategies for ovarian function and/or fertility preservation is mandatory in all premenopausal women with new cancer diagnosis (7–10), limited evidence exists on the actual use of these techniques (14). The PREFER study was designed to overcome this knowledge gap. This is important information to acquire for improving the oncofertility care and resource allocation in this area. Updated results of the PREFER study shows that the possibility of developing POI and/or subsequent impaired fertility worried most of premenopausal patients with breast cancer (93.7%). All patients concerned about the risk of developing POI accepted GnRHa use during chemotherapy as an option to preserve ovarian function. Among young women aged ≤40 years at diagnosis, approximately one out of 3 (34.6%) was interested in accessing the fertility unit but less than 1 out of 5 (18.2%) decided to undergo one or more of the offered cryopreservation options. The main reasons for refusal were prior completion of family planning, fear of delaying the initiation of antineoplastic treatments, refusal of further medicalization after complete counseling and lack of interest in the procedure after complete reproductive counseling.

An important issue in premenopausal patients facing breast cancer diagnosis and treatment is represented by the development of chemotherapy-induced POI with its subsequent infertility but also menopause-related consequences that include vasomotor symptoms, sexual dysfunction, body image chance, bone loss, cardiovascular risk and psychosocial issues (4). Therefore, informing premenopausal breast cancer patients about the risk of chemotherapy-induced POI is independent of future pregnancy desire (15, 16). The PREFER study aims to give important information also on this regard. Hence, unlike other studies that recruited only patients diagnosed at ≤40 years, our study allowed the inclusion of patients diagnosed between the age of 41 and 45 years to whom ovarian suppression with GnRHa is currently recommended as a standard strategy for reducing the risk of developing POI (15, 16).

In the PREFER study almost all patients (91% and 95% for patients diagnosed between 41 and 45 years and at ≤40 years, respectively) accepted the use of GnRHa during chemotherapy as a strategy to preserve ovarian function. A lower percentage of acceptance was demonstrated among the women enrolled in the American HOHO study (3.1% of patients aged ≤40 years) (17), and a slightly higher percentage in those included in the European HOHO study (24% of patients aged ≤40 years) (16). This difference can be partially explained by the publication, and subsequent recommendation from Italian guidelines (15), of the results of the Italian PROMISE-GIM6 study (18, 19) available since 2011. PROMISE-GIM6 study is the largest multicenter randomized study evaluating the efficacy and safety of GnRHa use during chemotherapy in premenopausal patients (aged less than 45 years) (18, 19). Moreover, another possible explanation for this difference is that the treatment with GnRHa during chemotherapy is reimbursed by the Italian National Health System (15). Notably, ovarian suppression with GnRHa is standard strategy for ovarian function preservation but it does not represent an alternative to cryopreservation techniques in young women interested in fertility preservation (7, 8, 10, 20). Premenopausal women with hormone receptor-positive breast cancer are candidate to adjuvant hormonal therapy for at least 5 years (7, 21, 22). In women at increased risk of disease recurrence including those exposed to prior chemotherapy use, ovarian function suppression with an aromatase inhibitor showed to be superior to either tamoxifen alone or tamoxifen combined with ovarian function suppression (23, 24). In premenopausal women developing chemotherapy-induced amenorrhea, administering an aromatase inhibitor alone may increase the risk of ovarian function recovery (25). Thus, starting GnRHa before chemotherapy could avoid the issues of defining ovarian function assessment following chemotherapy and the choice of the best endocrine therapy partner in this setting (24).

Besides the need for ovarian function preservation, young women with breast cancer may not have completed their family building plans at the time of diagnosis and might be interested in fertility preservation. Cryopreservation strategies are standard fertility preservation strategies (7–10). Preferably, ovarian tissue cryopreservation is proposed to women younger than 36 years, while oocyte and embryo cryopreservation is indicated up to the age of 40 years (9). Several barriers exist in discussing these options, including patient-related factors, cost of the strategies, lack of collaboration with a fertility unit, physicians’ inadequate knowledge of the different available strategies, or their concerns about the safety of pregnancy following breast cancer treatment (26). Although implementing a proper oncofertility program is crucial, our results highlight that only a minority of patients (18.2%) are finally motivated to undergo cryopreservation options. This is in line with other studies in both the US and Europe showing that, despite important concerns related to the development of this side effect, less than 10% of patients decide to undergo cryopreservation techniques (12, 16, 17). With a growing availability of efficacy and safety data, improved knowledge and financial coverage of these strategies, it is expected that the acceptance of such strategies will increase in the future (27–30). In fact, we found a higher percentage of patients accepting a cryopreservation technique (18.2%) compared to the previous analysis where only 12% of the patients accepted these surgical procedures (12). Nevertheless, the low number of patients undergoing cryopreservation strategies should be considered to improve the care in this setting. The creation of a solid oncofertility network with a hub and spoke regional distribution would be desirable (9, 31, 32). Thus, patients, from different oncology units that are interested in cryopreservation techniques can be referred to a smaller number of highly specialized fertility units in order to better optimize the access and success to these procedures. The collaborative network between oncology units and fertility centers might be useful also for counseling patients following anticancer treatment completion not only on pregnancy and conception but also about other reproductive issues including contraception and management of gynecological side effects of anticancer treatments (33). For achieving this goal, it is essential to establish a collaborative network between oncology units and fertility centers. Because of the many barriers existing in discussing ovarian and fertility preservation and building such network, we decided to extend the PREFER program to other Italian institutions. Multicenter data will become available in the near future and will help to further understand potential regional differences in oncofertility care and patients’ attitudes towards these issues.

Another important unresolved factor is the optimal timing for attempting pregnancy, especially in patients with hormone receptor-positive disease. Previous study demonstrated no difference in the access of reproductive counseling according to hormone receptor status, but a lower pregnancy rate among women with hormone receptor-positive disease (34, 35). We found a tendency for lower rates of access to cryopreservation strategies in patients with hormone receptor-negative disease as compared to those with hormone receptor-positive disease (10% *vs.* 19.4%). The longer period of anticancer treatment for patients with hormone-receptor positive disease with subsequent ovarian aging and need to postpone family planning might explain this attitude.

In conclusion, we demonstrated that the possible onset of chemotherapy-induced POI chemotherapy-induced and/or infertility worries the majority of newly diagnosed premenopausal breast cancer patients, and this appears to be particularly relevant in those with hormone receptor-positive disease. Use of GnRHa is a widely used and accepted method for ovarian function preservation. In women diagnosed at ≤40 years of age, approximately one out of 3 breast cancer patients accepted to undergo a counseling accepted to with a fertility specialist and less than 1 out of 5 decided to undergo a cryopreservation strategy. Our findings are relevant to improve the oncofertility counseling, for which it is essential to have a strong collaboration between oncologist and fertility specialist.

## Data Availability Statement

The raw data supporting the conclusions of this article will be made available by the authors, upon reasonable request to the corresponding author without undue reservation.

## Ethics Statement

The studies involving human participants were reviewed and approved by Comitato Etico Regionale, IRCCS Ospedale Policlinico San Martino. The patients/participants provided their written informed consent to participate in this study.

## Author Contributions

Conception and design: LDM, ML. Collection and assembly of data: EB, CM, LDM, ML. Data analysis and interpretation: EB, VF, LB, LDM, ML. All authors contributed to the article and approved the submitted version.

## Funding

The PREFER study is founded by “Regione Liguria – Assessorato alla Salute”, the Italian Association for Cancer Research (“Associazione Italiana per la Ricerca sul Cancro”, AIRC; MFAG 2020 ID 24698), the Italian Ministry of Health (5 x 1000 funds 2017). The funders had no role in study design, data collection and analysis, decision to publish, or preparation of the manuscript.

## Conflict of Interest

FP reports consulting or advisory role from Merck Sharp and Dohme; speakers’ bureau from Eli Lilly, Novartis; travel, accommodations, expenses from Eli Lilly, Takeda. CB reports advisory board, clinical trials and lecture fee from Novartis, Roche, Lilly and Pfizer, outside the submitted work. ML acted as a consultant for Roche, AstraZeneca, Novartis and Lilly, and received honoraria from Roche, Lilly, Novartis, Pfizer, Sandoz, and Takeda outside the submitted work.

The remaining authors declare that the research was conducted in the absence of any commercial or financial relationships that could be construed as a potential conflict of interest.
